# Successful hemostasis of a parapapillary diverticular hemorrhage by the retroflexion technique in the descending part of duodenum

**DOI:** 10.1055/a-1931-4161

**Published:** 2022-09-30

**Authors:** Kurato Miyazaki, Motohiko Kato, Motoki Sasaki, Teppei Masunaga, Atsushi Nakayama, Takanori Kanai, Naohisa Yahagi

**Affiliations:** 1 Division of Gastroenterology and Hepatology, Department of Internal Medicine, Keio University School of Medicine, Tokyo, Japan; 2 Division of Research and Development for Minimally Invasive Treatment, Cancer Center, Keio University School of Medicine, Tokyo, Japan

In endoscopic procedures, the retroflexion technique is often useful to overcome difficult situations 1 2 3 4 5 . Here, we report a successful hemostasis case of parapapillary diverticular hemorrhage by the retroflexion technique in the descending part of duodenum.

An 82-year-old man was admitted to our hospital for treatment of tarry stools and underwent an urgent endoscopy. Bleeding from a parapapillary diverticulum was suspected, but the bleeding point could not be identified by either a forward-viewing or side-viewing endoscope. It was judged that spontaneous hemostasis had been achieved, but re-bleeding was suspected because anemia progressed gradually. A few days later, endoscopy was performed again ( Video 1 ).

**Video 1** Successful hemostasis of a parapapillary diverticular hemorrhage by the retroflexion technique in the descending part of the duodenum.


Like the initial endoscopy, we could see blood flow from the parapapillary diverticulum, but the bleeding point was invisible ( Fig. 1 ). We previously reported a successful case in which endoscopic mucosal resection for a duodenal adenoma was performed using the retroflexion technique 1 , and we presumed that a similar approach might be helpful. We changed the normal tip hood to a therapeutic tapered hood to facilitate entry into the diverticulum and inverted the endoscope at the inferior duodenal angle ( Fig. 2 ). Consequently, we could successfully approach the diverticulum and clearly identify the bleeding point ( Fig. 3, Fig. 4 ). Because of the narrow working space, unnecessary clip placement had to be avoided. Therefore, a re-openable clip was used to ensure that the bleeding point was definitely grasped before the clip was placed. Effective hemostasis was achieved with only one clip ( Fig. 5 ), and the patient was discharged without re-bleeding thereafter.

**Fig. 1:**
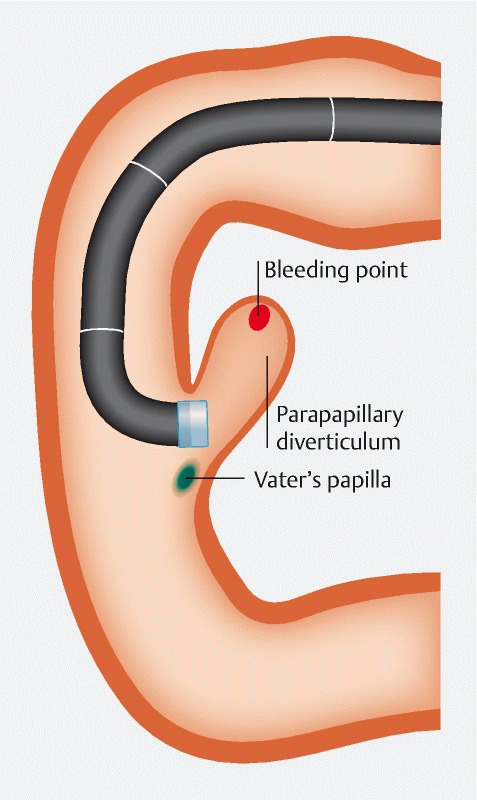
Illustration of the forward-viewing approach to the parapapillary diverticular hemorrhage. We could not clearly identify the bleeding point.

**Fig. 2:**
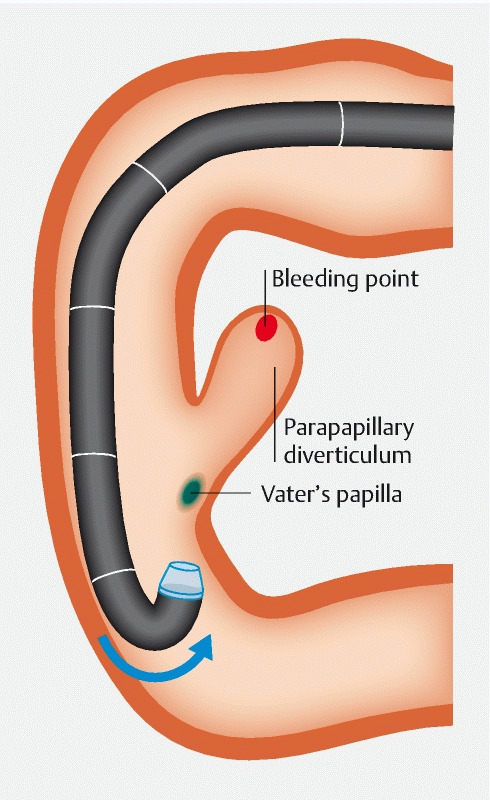
Illustration of inversion maneuvering at the inferior duodenal angle. We carefully inverted the endoscope at the inferior duodenal angle.

**Fig. 3:**
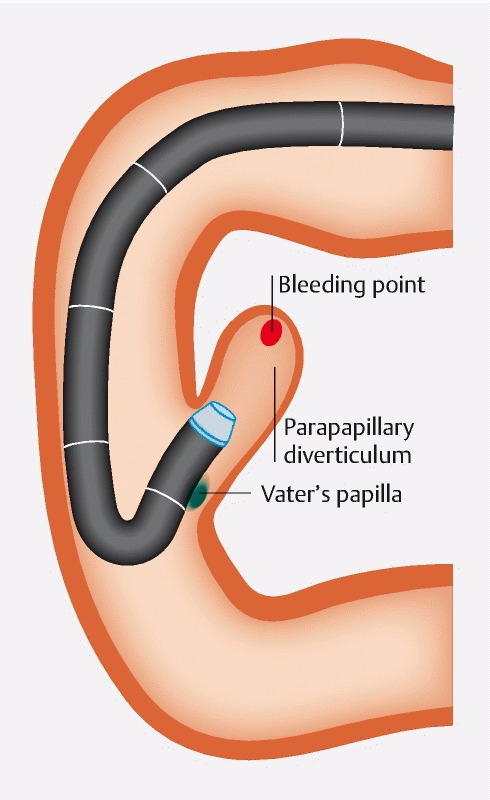
Illustration of the retroflexion technique at the parapapillary diverticulum with tapered hood. We were able to enter the parapapillary diverticulum and identify the bleeding point.

**Fig. 4:**
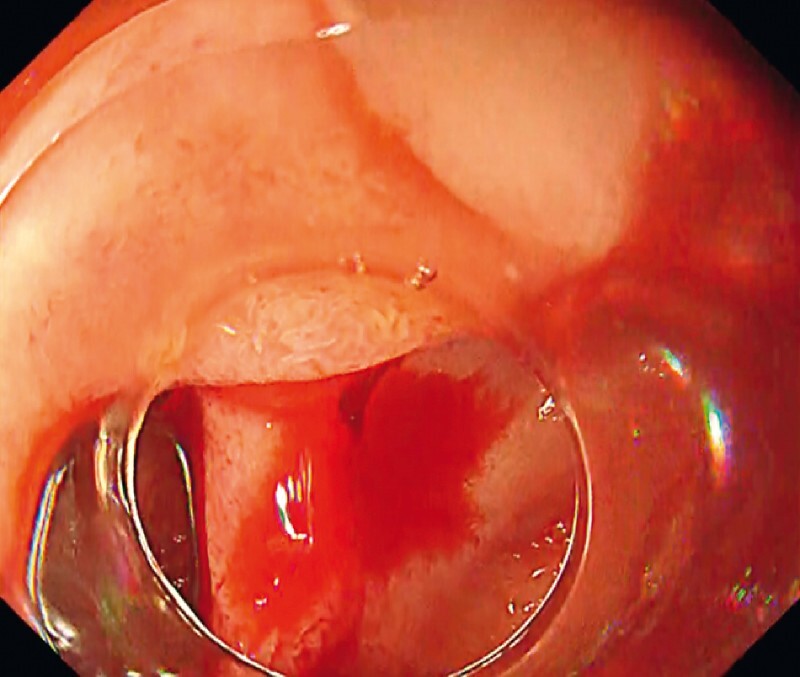
White light imaging of the bleeding point. We could clearly identify the bleeding point by the retroflexion technique.

**Fig. 5:**
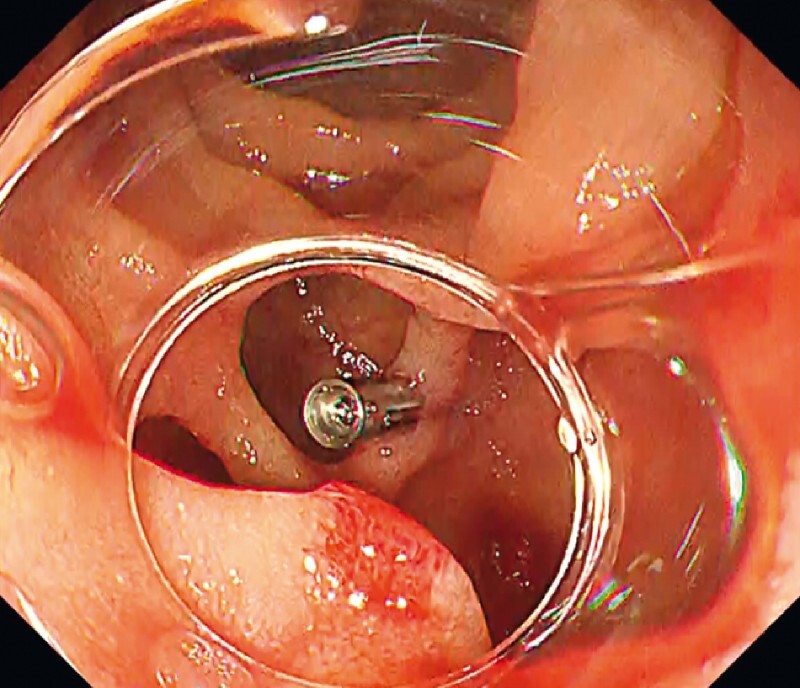
White light imaging of the parapapillary diverticulum after clipping. Effective hemostasis was achieved with only one clip. Vaters papilla was observed on the anal side of the diverticulum.

In this case, because the parapapillary diverticulum opened toward the anal side, the bleeding point was invisible by the forward-viewing approach. However, the retroflexion technique dramatically improved the visibility of the bleeding point. The retroflexion technique may be one way to overcome such difficult situations, although careful endoscopic maneuvering is required since the duodenal lumen is narrow.

Endoscopy_UCTN_Code_CCL_1AB_2AD_3AZ

## Notes

Endoscopy E-Videos

https://eref.thieme.de/e-videos
*Endoscopy E-Videos* is an open access online section, reporting on interesting cases and new techniques in gastroenterological endoscopy. All papers include a high quality video and all contributions are freely accessible online. Processing charges apply (currently EUR 375), discounts and wavers acc. to HINARI are available. This section has its own submission website at https://mc.manuscriptcentral.com/e-videos

## References

[1] Masunaga T Kato M Retroflexion technique in the descending part of the duodenum for endoscopic mucosal resection Dig Endosc 2021 33 e45 e46 10.1111/den.13920 33506590

[2] Tanaka H Oka S Tanaka S The utility of a novel colonoscope with retroflexion for colorectal endoscopic submucosal dissection Endosc Int Open 2019 7 E130 E137 10.1055/a-0810-0567 30705943 PMC6336463

[3] Liu S Li Y Yang H Retroflexion-assisted endoscopic mucosal resection: a useful and safe method for removal of low rectal laterally spreading tumors Surg Endosc 2016 30 139 146 10.1007/s00464-015-4173-2 25807863

[4] Fujihara S Kobara H Mori H Comparison of retroflexed and forward views for colorectal endoscopic submucosal dissection Int J Med Sci 2015 12 450 457 10.7150/ijms.11930 26078705 PMC4466509

[5] Rex D K Vemulapalli K C Retroflexion in colonoscopy: Why? Where? When? How? What value? Gastroenterology 2013 144 882 883 10.1053/j.gastro.2013.01.077 23499952

